# Do We Use Os-Artificial?

**Published:** 1869-03

**Authors:** 


### DO WE USE OS-ARTICIAL?

Yes, in almost all cases where any thing else is called for than gold. For temporary purposes we regard it, all things considered, as the best material now in use. Its efficiency depends much upon the manner of using it. Like other things it must be properly manipulated, to secure the best results. The first point, after having a good material, is the thorough mixing or kneading, which must be done quickly, and introduced before solidification begins. The mass when introduced should be of that consistence in which the watery appearance upon the surface does not appear.

The next important point is retaining the filling dry till the material is thoroughly hardened. This will require from ten to fifteen minutes; indeed, it is better to protect the surface from moisture for a much longer time, which may be done by a coating of softened adhesive wax, requesting the patient to retain it as long as possible; after this a better finish may be given to the filling than it was possible to make when it was introduced.

By pursuing the plan indicated, fillings may be made of this material that will remain for several years; their durability will however, depend much upon the exposure in mastication; for fillings upon the masticating surfaces of the molars and bicuspids, it is of little value; especially in small and medium sized cavities. T.

